# Cytokine-mediated protection of human dendritic cells from prostate cancer-induced apoptosis is regulated by the Bcl-2 family of proteins

**DOI:** 10.1054/bjoc.2000.1289

**Published:** 2000-07-24

**Authors:** G Pirtskhalaishvili, G V Shurin, C Esche, Q Cai, R R Salup, S N Bykovskaia, M T Lotze, M R Shurin

**Affiliations:** Department of Urology, Surgery, University of Pittsburgh Medical Center and University of Pittsburgh Cancer Institute, Pittsburgh, PA 15213, USA; Allegheny-Zinger Research Institute, Pittsburgh, PA 15212, USA; University of South Florida, Tampa, FL 33620, USA

**Keywords:** prostate cancer, dendritic cells, immunosuppression, apoptosis, Bcl-2

## Abstract

Prostate cancer is the most common cancer in men in the United States, and second in cancer-induced mortality. It is likely that tumour-induced immunosuppression is one of the reasons for low treatment efficacy in patients with advanced prostate cancer. It has been recently demonstrated that prostate cancer tissue is almost devoid of dendritic cells (DC), the major antigen-presenting cells responsible for the induction of specific antitumour immune responses. In this study, we have tested the hypothesis that prostate cancer induces progressive suppression of the DC system. We found that co-incubation of human DC with three prostate cancer cell lines led to the high levels of premature apoptosis of DC, which were significantly higher than in DC cultures co-incubated with normal prostate cells or blood leucocytes. Stimulation of DC for 24 hours with CD40 ligand (CD154), IL-12 or IL-15 prior to their co-incubation with prostate cancer cells resulted in a significant increase in DC survival in the tumour microenvironment. Furthermore, activation of DC with these cytokines was also accompanied by increased expression of the anti-apoptotic protein Bcl-x _L_ in DC, suggesting a possible mechanism involved in DC protection from apoptotic death. In summary, our data demonstrate that prostate cancer induces active elimination of DC in the tumour microenvironment. Stimulation of DC by CD154, IL-12 or IL-15 leads to an increased expression of the anti-apoptotic protein Bcl-x _L_ and increased resistance of DC to prostate cancer-induced apoptosis. These results suggest a new mechanism of tumour escape from immune recognition and demonstrate the cytokine-based approaches which might significantly increase the efficacy of DC-based therapies for cancer. © 2000 Cancer Research Campaign


*British Journal of Cancer* (2000) **83**(4), 506–513
© 2000 Cancer Research Campaign
doi: 10.1054/ bjoc.2000.1289, available online at http://www.idealibrary.com on IDEAL®

### Cytokine-mediated protection of human dendritic cells from prostate cancer-induced apoptosis is regulated by the Bcl-2 family of proteins

**G Pirtskhalaishvili[1], GV Shurin[2], C Esche[2], Q Cai[2], RR Salup[4], SN Bykovskaia[3], MT Lotze[2] and MR Shurin[2]**

Department of [1]Urology and [2]Surgery, University of Pittsburgh Medical Center and University of Pittsburgh Cancer Institute, Pittsburgh PA 15213, USA; [3]Allegheny-Zinger Research Institute, Pittsburgh PA 15212, USA; [4]University of South Florida, Tampa FL 33620, USA

**Summary** Prostate cancer is the most common cancer in men in the United States, and second in cancer-induced mortality. It is likely that tumour-induced immunosuppression is one of the reasons for low treatment efficacy in patients with advanced prostate cancer. It has been recently demonstrated that prostate cancer tissue is almost devoid of dendritic cells (DC), the major antigen-presenting cells responsible for the induction of specific antitumour immune responses. In this study, we have tested the hypothesis that prostate cancer induces progressive suppression of the DC system. We found that co-incubation of human DC with three prostate cancer cell lines led to the high levels of premature apoptosis of DC, which were significantly higher than in DC cultures co-incubated with normal prostate cells or blood leucocytes. Stimulation of DC for 24 hours with CD40 ligand (CD154), IL-12 or IL-15 prior to their co-incubation with prostate cancer cells resulted in a significant increase in DC survival in the tumour microenvironment. Furthermore, activation of DC with these cytokines was also accompanied by increased expression of the anti-apoptotic protein Bcl-$x_L$ in DC, suggesting a possible mechanism involved in DC protection from apoptotic death. In summary, our data demonstrate that prostate cancer induces active elimination of DC in the tumour microenvironment. Stimulation of DC by CD154, IL-12 or IL-15 leads to an increased expression of the anti-apoptotic protein Bcl-$x_L$ and increased resistance of DC to prostate cancer-induced apoptosis. These results suggest a new mechanism of tumour escape from immune recognition and demonstrate the cytokine-based approaches which might significantly increase the efficacy of DC-based therapies for cancer. © 2000 Cancer Research Campaign

**Keywords:** prostate cancer; dendritic cells; immunosuppression; apoptosis; Bcl-2

Prostate cancer is the most common cancer in the United States, where about 180 000 men were expected to be diagnosed with prostate cancer in 1999. Approximately 40 000 men die of this disease every year in the US, according to American Cancer Society estimation (Landis et al, 1999). Though localized prostate cancer can be treated with radical surgery or radiation therapy, there is no curative treatment for advanced disease. In addition, one third of radically treated patients ultimately experience a relapse. Endocrine therapy offers only temporary relief, and chemotherapy is largely ineffective. Prostate cancer shortens life expectancy of men by approximately 4–5 years compared to the age-matched general population, and approximately one man out of 6 has a lifetime risk of developing prostate cancer. This makes a better understanding of prostate cancer's nature and the development of new treatment methods crucial.

The immunologic status of prostate cancer patients has been evaluated during the last several years. Immunosuppression is noted in relatively confined prostate tumours, and a number of studies demonstrated a significant inhibition of the immune system at advanced stages in prostate cancer patients (Herr, 1980; Ivshina et al, 1995; Healy et al, 1998; Salgaller et al, 1998). However, the exact nature and the level of immunodeficiency in these patients as well as its clinical, biological and prognostic significance are still controversial.

*Received 3 December 1999*
*Revised 27 March 2000*
*Accepted 10 April 2000*

*Correspondence to:* MR Shurin

During the last decade a great deal of attention has been paid to the role of dendritic cells (DC) in the development of immune responses. First described in 1973 (Steinman and Cohn, 1973), DC originate from CD34+ progenitor cells in the bone marrow. Within non-lymphoid tissue these cells apparently present as immature DC with the capacity to recognize, take up, and process antigen. After acquiring antigen, they can migrate to the lymph nodes and present the antigen to the T cells, stimulating their differentiation and clonal expansion. Thus, as antigen presenting cells (APC), DC play a crucial role in the development of specific antitumour immune responses (Shurin, 1996). In contrast, suppression of the DC system may effectively eliminate the induction and development of a specific immune reaction. Indeed, it has been recently shown that DC may undergo apoptosis in vivo and in vitro after contact with tumour (Esche et al, 1999; Shurin et al, 1999). Decreased number of DC has been demonstrated during the progression of melanoma (Toriyama et al, 1993; Stene et al, 1988), and increased DC infiltration into primary tumour lesions has been associated with better survival (Becker, 1992; Lotze, 1997).

Prostate cancer has also been studied for the presence of DC infiltrate. It was shown that the number of DC decreased in the human prostatic cancer tissue, in comparison to normal prostatic and benign prostate hyperplasia (BPH) tissues (Davidson et al, 1997; Troy et al, 1998). In addition, reversed correlation between degree of differentiation and the number of DC within the prostate was found: the higher the anaplastic rate, the fewer DC were found in the prostate cancer tissue. One of the explanations of this finding is that prostate cancer microenvironment effectively suppresses or eliminates DC in situ, thus diminishing the immunologic response of the host and allowing tumour to progress (Shurin, 1999).

When it was generally accepted that DC play a major role in the induction and development of specific immunologic responses, several clinical trials were initiated which used DC-based immunotherapies to treat cancer patients, including patients with prostatic carcinomas. For instance, Salgaller et al (1998) treated prostate cancer patients using DC pulsed with prostate membrane specific antigen (PMSA)-associated peptides. The results of this and similar clinical trials were quite optimistic. However, presence of patients with limited or no responses suggests that immunobiology of DC in in vivo tumour microenvironment might be different from our expectations and indicates the actual need for the improvement of modern DC-based immunotherapies. We hypothesized that one of the reasons for the limited efficacy of the immune responses in treated patients may be the prostate cancer-induced suppression and elimination of DC.

Premature apoptosis of DC, induced by different tumour cell lines, has been described early (Esche et al, 1999). However, signal transduction pathways regulating this phenomenon as well as mechanisms of DC protection were not yet investigated. It has been recently demonstrated that cytokines may increase survival of immune cells, including DC, in cultures and that this effect was mediated by the up-regulated expression of anti-apoptotic proteins which belong to the Bcl-2 family of proteins. For example, CD40L (CD154) has been reported to stimulate DC maturation and survival in vitro (Bjorck et al, 1997; Mackey et al, 1998). Interestingly, CD40 ligation on other cell types was associated with increased expression of anti-apoptotic proteins Bcl-2 and Bcl-$x_L$ (Ning et al, 1996; Alam et al, 1997; Akifusa et al, 1998). Interleukin 12 (IL-12), a proinflammatory cytokine with a wide spectrum of functions, enhances the survival of hematopoietic progenitor cells (Ploemacher et al, 1993; Bellone and Trinchieri, 1994). Interleukin-15 (IL-15), a cytokine with similar to IL-2 activities, was also shown to increase survival of NK cells (Carson et al, 1997) and lymphocytes (Bulfone-Paus et al, 1997; Lorenz et al, 1997). These effects of IL-15 were accompanied by up-regulation of expression of anti-apoptotic protein Bcl-2 in lymphocytes (Carson et al, 1997; Lorenz et al, 1997). Thus, cytokines may regulate differentiation and survival of immune cells. However, their effect on immune cell survival in the tumour microenvironment was not evaluated.

The aims of this study were to (i) determine whether human prostate cancer may cause apoptosis of human DC, (ii) evaluate possible mechanisms of DC death and (iii) examine the role of cytokines IL-12 and IL-15 in the protection of DC from tumour-induced cell death. Using several different approaches, we have demonstrated that three different prostate cancer cell lines caused apoptotic death of DC during co-incubation. CD40 ligation on DC induced production of IL-12 and IL-15, which both increased resistance of DC to prostate cancer-induced apoptosis by up-regulating expression of Bcl-2 proteins.

### MATERIALS AND METHODS

### Cells

Prostate cancer cell lines, LNCaP, DU-145 and PC-3, were cultured in RPMI 1640 medium (GIBCO, Grand Island, NY) supplemented with 2 mM L-glutamine, 100 units/ml penicillin, 100 μg/ml streptomycin, 1 mM sodium pyruvate, 0.1 mM nonessential amino acids, and 5% heat inactivated fetal bovine serum (FBS) (all from GIBCO). Human normal prostate epithelial cells (PrEC) were obtained from Clonetics Corporation (Walkersville, MD) and maintained in special medium according to the manufacturer's recommendations.

Dendritic cells were generated from CD14+ monocytes obtained from human peripheral blood of healthy volunteers. After gradient separation on HistoPrep medium (1.077 g/ml; Sigma, St. Louis, MO), adherent cells were isolated from peripheral blood mononuclear cells (PBMC) on plastic (37°C, 2 hours) and cultured in tissue flasks in complete medium, consisting of AIM-V (GIBCO), 2.5% heat inactivated human AB serum (Sigma), 1 μg/ml indomethacin (Sigma) and 50 μM N-methyl-L-arginine (Alexis, San Diego, CA). 1000 U/ml *rh*GM-CSF and 1000 U/ml *rh*IL-4 (a gift from Schering-Plough Research Institute, Kenilworth, NJ) were added to the cultures every 3 days. Non-adherent cultured DC were harvested 7–10 days later and DC phenotype was confirmed by flow cytometry. Cultured cells were CD14-, CD3-, CD20-, HLA-DR+, CD80+, CD86+, CD40+ suggesting a common phenotype of cultured monocyte-derived DC.

### Experimental design

Cultured DC were collected, purified from dead cells (usually <5–10%), counted and incubated with human prostate cancer cell lines LNCaP, DU-145 or PC-3 in 6-well plates. Cells were separated using membrane inserts with 0.4 μm pore size. Five to 10 × $10^5$ DC were placed in wells with 2 ml complete medium. One to 2 × $10^6$ prostate cancer cells resuspended in 2 ml of medium were placed in the inserts. As controls, DC were co-incubated with normal human prostate epithelial cells PrEC, human PBMC or tumour medium alone. DC were collected 24 and 48 hours later and apoptosis was assessed by the morphological criteria, TUNEL staining and Annexin V binding assay.

To evaluate whether cytokines protect DC from tumour-induced apoptosis, cultured DC were stimulated with IL-12, IL-15 and CD40 ligand (CD154) for 24 hours before the co-incubation with LNCaP prostate cancer cells. One hundred ng/ml of recombinant human IL-12 (*rh*IL-12, a gift from Hoffman-La Roche Inc., Nutley, NJ), and 300 ng/ml of recombinant human IL-15 (*rh*IL-15, Immunex Corp., Seattle, WA) were used in these studies. Two methods were used for the stimulation of CD40 on the surface of DC. In one case, L-cells (murine fibroblasts) transfected with human CD40 ligand (CD40L/L-cells) (Arpin et al, 1995) were used to activate DC. DC were incubated with adherent CD40L/L-cells for 24 hours prior to their co-incubation with prostate cancer cells. DC incubated with nontransfected L-cells served as controls. In the second case, mouse anti-human CD40 antibodies (1 μg/ml, Upstate Biotechnology, Lake Placid, NJ) were used to stimulate DC in cultures for 24 hours followed by the co-incubation with tumour cells. DC stimulated with mouse IgG (Sigma) at the same concentration provided a control for these experiments.

### Assessment of apoptosis

#### *Morphologic evaluation*

Dendritic cells were harvested after co-incubation with tumour cells and placed on microscope slides using a standard Cytospin

© 2000 Cancer Research Campaign 

centrifuge (300 rpm, 5 min) (Shandon Lipshaw, Pittsburgh, PA). Cells were stained with a LeukoStat Stain Kit (Fisher Scientific, Pittsburgh, PA) and the percentage of apoptotic cells was assessed quantitatively using morphologic criteria, including condensation of chromatin, reduction in nuclear size, shrinkage of total cell volume, increased cell density, and a formation of apoptotic bodies (Kerr et al, 1972).

### *Annexin V assay*

DC were collected and double-stained with FITC-conjugated Annexin V (PharMingen, San Diego, CA) and propidium iodide (PI) (Sigma). Annexin V was added according to the manufacturer's recommendations. PI was used at a final concentration of 10 μg/ml. The percentage of cells undergoing apoptosis in a quantitative manner was determined as percentage of Annexin V positive PI negative cells. Samples were analysed by the FACScan flow cytometer with LYSYS II software package (Becton Dickinson, San Jose, CA).

### *TUNEL staining*

Cells were collected, washed, counted, and $10^6$ cells/ml were fixed consecutively in 4% formaldehyde and 80% ethanol. Fixed cells were placed on glass slides using Cytospin centrifuge. TdT-FragEl DNA fragmentation detection kit (Oncogene Research Products, Cambridge, MA) was used for terminal deoxynucleotidyl transferase and nick translation (TUNEL) assay to determine the apoptosis. TUNEL positive and negative cells were counted at least in ten different fields and the number of apoptotic cells was expressed as the percentage of TUNEL positive cells.

### RT–PCR

Total RNA from harvested DC was extracted by Quanidium method with RNAZol B (Biotek Lab. Inc., Houston, TX) following the procedure recommended by the manufacturer. Briefly, homogenized samples were mixed with RNAZol B, incubated on ice for 5 min and mixed with 1/10 volume of chloroform. Then samples were vigorously shaken for 15 sec, incubated on ice for 5 min and centrifuged at 12 000 ***g*** for 15 min at 4°C. Aqueous phase was transferred into an equal volume of isopropanol and incubated at –20°C for at least 15 min. RNA was then precipitated by centrifugation and washed with ethanol. The dried pellet was dissolved in diethylpyrocarbonate (DEPC)-treated water and the amount of RNA was determined by a spectrophotometric reading at 260 nm. Synthesis of DNA was carried out in a 50 mM Tris-HCl buffer (pH 8.3) containing 75 mM KCl, 3 mM $MgCl_2$, 10 mM DTT, 40 U/ml RNAse inhibitor, 200 U/ml Molony Murine Leukemia Virus (MMLV)-Reverse Transcriptase, 1.25 mM of each dATP, dTTP, dGTP, dCTP, 20 μg/ml oligo-dT and isolated RNA samples, which were preheated at 70°C and cooled on ice. The reaction mixture was incubated at 37°C for one hour followed by a brief heat inactivation of the enzyme. The synthesized cDNA was subjected to PCR amplification with human β-actin primers to verify the quality of RNA/cDNA preparation: 5′-GCATCGTCACCAACTGGGACGAC-3′ and 5′-ATTTGCGGTGGACGATGGAGGGGC-3′. All samples tested for the *h*IL-12 mRNA expression were confirmed to be β-actin positive. To verify that the PCR product was not amplified from residual DNA left in RNA samples, RNA preparations from all samples without cDNA step (RT negative control) were subjected to β-actin PCR amplification. The following primers for *h*IL-12 p40 were used: 5′-ATTGAGGTCATGGTGGATGC-3′ and 5′-AATGCTGGCATTTTTGCGGC-3′. To perform a PCR, synthesized cDNA (100 ng or more RNA equivalent cDNA) was added to a final volume of 100 μl 10 mM Tris-HCl buffer (pH 8.3) containing 50 mM KCl, 1.5 mM $MgCl_2$, 200 μM dNTP, 0.2 μM primer and 2.5 U/ml Taq DNA polymerase (Perkin Elmer, Norwalk, CT). The reaction was performed in a thermal cycler (Perkin Elmer) at 94°C 1 min, 60°C (for β-actin) or 55°C (for *h*IL-12 p40) 1 minute, and 72°C 1 minute each cycle, for the total of 30 to 35 cycles, with an extension of 10 minutes at 72°C after the last cycle. To detect the PCR product, 40 μl of PCR samples were loaded on an agarose gel at the appropriate concentration followed by electrophoresis.

Human IL-15 was detected by RT–PCR using primers: 5′-AGCCAACTGGGTGAATGTAA-3′ and 5′-TTGCATCCAGAT-TCTGTTAC-3′ with similar conditions as described above for the *h*IL-12-related procedures.

The comparison and analysis of mRNA expression was done by volume quantitation using BioRad Densitometer. Sample/β-actin ratio was first calculated for each sample and then used for comparison. Thus, increased expression of mRNA is presented as a percentage from the control (non-stimulated) value.

### Western blot

The expression of anti-apoptotic protein Bcl-$x_L$ in DC was assessed using Western blot technique. Cells were collected, washed in PBS and homogenized in a lysing buffer. The homogenate was then centrifuged at 12 000 ***g*** for 15 min at 4°C. The protein concentration in the supernatant was determined by the Bradford method, using BioRad protein kit (Bio-Rad Laboratories, Hercules, CA). Each sample was denaturated for 5 min at 100°C in a sample buffer. Equal amounts of protein were loaded for each sample and electrophoretically separated on 16.5% SDS-PAGE followed by transfer to a nitrocellulose membrane. The membrane was blocked with 0.2% nonfat milk (BioRad) and 0.1% Tween-20 (Fisher) in 20 mM Tris-saline buffer at pH 7.2. Bcl-$x_L$ protein was detected using specific rabbit primary antibodies (Oncogene Research Products) with the final concentration of 2.5 μg/ml, and donkey anti-rabbit secondary antibodies (1: 5000 dilution, Amersham Pharmacia Biotech Inc., Piscataway, NJ). The membrane was processed and treated with chemiluminescence reagents (Tropix, Medford, MA). The bands were visualized on a Kodak film exposed to the membrane to detect chemiluminescence signals. The comparison and analysis of protein expression was performed using BoiRad Densitometer as described above.

### Statistical analysis

Chi-square analysis was performed to evaluate the significance of differences between the experimental groups in the Annexin V staining assays, when discrete data were presented. For a single comparison of two groups, the Student's *t*-test (SigmaStat Statistical Software, SPSS, Chicago, IL) was used. If data distribution was not normal, Mann-Whitney rank sum test was employed. For all analysis, the level of significance was set at a probability of 0.05 to be considered significant. Data are presented as the mean ± SEM.

 © 2000 Cancer Research Campaign

### RESULTS

### Co-incubation of prostate cancer cells and DC resulted in apoptotic death of DC

DC were co-incubated with LNCaP, DU-145 and PC-3 cells across the membrane with 0.4 μm pore size, collected, and the levels of apoptosis were determined after 24 and 48 hours. DC co-incubated with PrEC, PBMC and tumour medium served as controls. The evaluation of DC survival incubated with prostate cancer cells or control cells demonstrated a marked difference in resulted cell morphology, including condensed cytoplasm and nuclei, degradation of chromogen and formation of apoptotic bodies in DC cultures co-incubated with tumour cells (Figure 1). According to the morphologic evaluation, DC incubated with LNCaP, DU-145, PC-3, PBMC and tumour medium had an apoptotic rate of 23.65 ± 2.90%, 27.77 ± 4.61%, 22.32 ± 2.51%, 7.41 ± 1.30% and 7.25 ± 1.03%, respectively (Figure 2). Statistical analysis of data confirmed significantly lower numbers of apoptotic cells in DC cultures co-incubated with PrEC or PBMC in comparison with DC cultures co-incubated with LNCaP ($P < 0.001$), DU-145 ($P < 0.01$) and PC-3 ($P < 0.001$) tumour cell lines. Furthermore, neither proliferation of tumour cells nor depletion of nutrients within the culture medium was responsible for the DC apoptosis as was shown in additional experiments with multiple replacement of tumour cells. We next confirmed these results using Annexin V binding assay: levels of DC apoptosis were 30.89 ± 4.10%, 18.95 ± 10.81%, 24.62 ± 4.70%, 8.26 ± 1.78% and 5.27 ± 1.90% in DC cultures co-incubated with LNCaP, DU-145, PC-3 PBMC and medium, respectively. Prostate cancer cell-induced suppression of DC survival in cultures was statistically significant as compared to control value ($P < 0.001$ in all cases) (Figures 2 and 3). Analysis of TUNEL staining in different DC cultures confirmed that pre-incubation of DC with prostate cancer cells resulted in a significant increase in TUNEL-positive cell numbers in these cultures (Figure 1). Each experiment was repeated at least twice. Taken together, these data demonstrated that prostate cancer cells significantly shortened the life-span of DC in cultures by increasing the levels of apoptotic death in these cells.

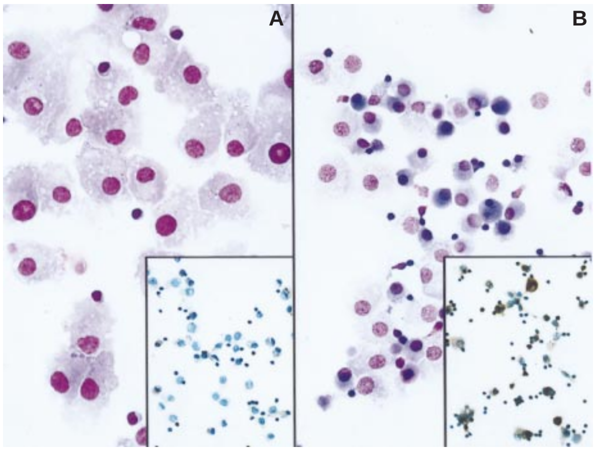

**Figure 1** Prostate cancer cells cause apoptosis of human dendritic cells. Incubation of human cultured DC with LNCaP cells (**B**) caused morphological alterations of DC which correspond to the features of apoptotic cell death. Incubation of DC with PBMC, instead of tumour cells, served as a control (**A**). DC were harvested after 48 hours of co-incubation, fixed, stained with Eosin Y and Methylene blue and analysed by light microscopy (× 400), as described in Materials and Methods. TUNEL staining of DC confirmed that co-incubation of DC with LNCaP cells resulted in a significant increase in the levels of DC apoptosis, as can be seen as brown (TUNEL+) cells in insets

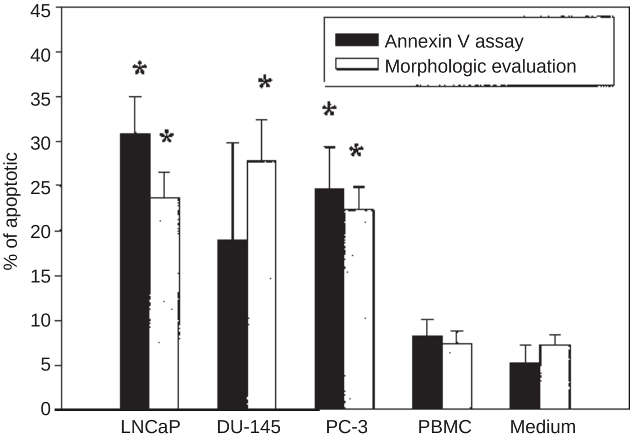

**Figure 2** Three different prostate cancer cell lines cause apoptosis of human dendritic cells. The levels of apoptotic death in DC cultures were significantly increased after 48 hours of co-incubation with different prostate cancer cells when compared with control DC cultures co-incubated with PrEC, PBMC or tumour medium. DC were generated from human adherent PBMC, co-incubated with tumour or control cells, harvested, washed and analysed. The percentage of apoptotic cells was determined by the morphological features and Annexin V binding assay, as described in Materials and Methods.*$P < 0.01$ compare to the control group

### CD40 ligation protected DC from prostate cancer-induced apoptosis

Since it has been shown early that CD154 might prolong DC survival in cultures, we have examined the influence of CD40 ligation on DC survival in the prostate cancer microenvironment in vitro. Incubation of human DC with CD154 (CD40L)-transfected L-cells for 24 hours before the co-incubation with LNCaP cells, decreased the morphological appearance of apoptosis from 18.39 ± 1.63% in DC cultures pre-incubated with non-transfected L-cells to 9.55 ± 1.60% ($P < 0.01$). Analysis of an Annexin V binding assay revealed the similar data and confirmed the significant decrease of prostate cancer-induced apoptotic rate by 16% ($P < 0.01$) in cultured DC after CD40 ligation.

The alternative way of stimulation of CD40 receptor using anti-CD40 antibodies confirmed our results. For instance, activation of DC with anti-CD40 antibodies for 24 hours followed by the co-incubation with LNCaP prostate cancer cells resulted in 13.49 ± 2.80% of apoptosis in DC, whereas pre-incubation of DC with isotype-matched control IgG followed by the co-incubation with LNCaP cells caused apoptosis in 21.89 ± 5.51% of DC, as was assessed by Annexin V binding assay ($P < 0.001$). All experiments were repeated at least twice with the same effect. Thus, our data demonstrated that ligation of CD40 receptors on DC led to DC activation and increased their resistance to prostate cancer-induced apoptosis.

### Cytokines IL-12 and IL-15 protected DC from prostate cancer-induced apoptosis

To determine the resistance of cytokine-activated DC to the induction of cell death in the tumour microenvironment, we have

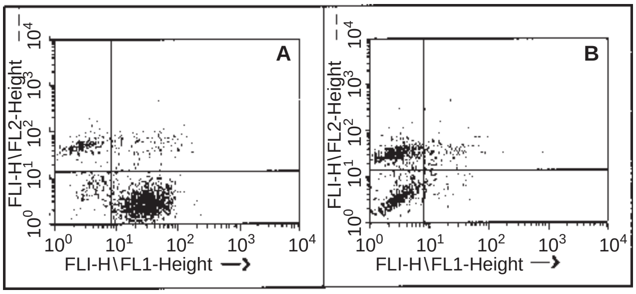

**Figure 3** FACScan detection of apoptotic changes in the membranes of human dendritic cells after co-incubation with LNCaP cells. Cultured human DC were co-incubated with LNCaP cells (**A**) or control PBMC (**B**) separated by a membrane with 0.4 μm pore size for 48 hours. DC were harvested, washed, and the level of apoptotic death was determined using Annexin V (x axis) and propidium-iodide (PI) (y axis) binding assay as described in Materials and Methods. Annexin-positive (apoptotic) and PI negative (non-necrotic) cells are localized in the right lower quadrant

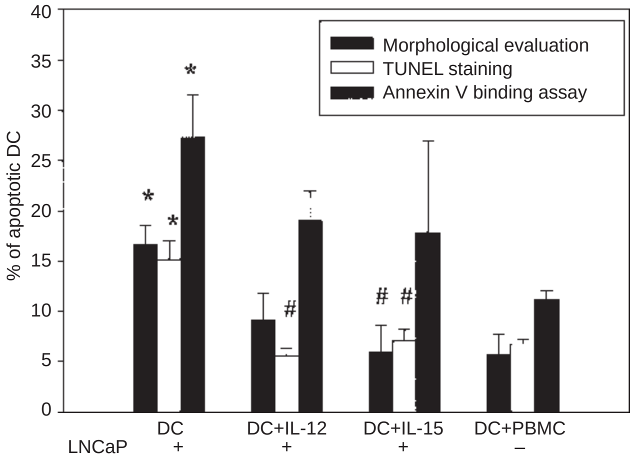

**Figure 4** Activation of dendritic cells with cytokines increases their resistance to tumour-induced apoptosis. Cultured human DC were treated with cytokines IL-12 and IL-15 for 24 hours before the co-incubation with prostate cancer cells. DC were then collected, washed and the levels of apoptosis were assessed by the morphologic evaluation, TUNEL staining and an Annexin V binding assay, as described in Materials and Methods. Pretreatment of DC with cytokines significantly decreased levels of prostate cancer-induced apoptosis of DC.*$P < 0.01$ and #$P < 0.05$ compared to the control group examined whether cytokines IL-12 or IL-15 protect DC from prostate cancer-induced apoptosis. Cultured DC were stimulated with *rh*IL-12 (100 ng/ml) or *rh*IL-15 (300 ng/ml) for 24 hours before the co-incubation with LNCaP cells. Cell viability was assessed by the morphologic examination, TUNEL staining and Annexin V binding assay.

Morphologic evaluation of DC survival demonstrated that levels of DC apoptosis in cultures co-incubated with PBMC (control), LNCaP alone or LNCaP following IL-12 or IL-15 stimulation were 5.51 ± 2.12%, 16.58 ± 2.01%, 9.06 ± 2.70%, and 5.80 ± 2.75%, respectively. These data showed that cytokines IL-12 and IL-15 significantly decreased the level of tumour-induced apoptosis in DC ($P < 0.05$ for IL-12 and $P < 0.01$ for IL-15) (Figure 4). Similarly, TUNEL staining demonstrated the tumour-induced apoptotic rate of 6.59 ± 0.48%, 15.12 ± 1.86%, 5.50 ± 0.77%, and 6.92 ± 1.14% in the same DC cultures, respectively ($P < 0.001$ for IL-12 and $P < 0.01$ for IL-15). Annexin V binding assays further confirmed these data: DC apoptotic rates were 11.02 ± 0.90%, 27.26 ± 4.26%, 18.97 ± 2.95%, and 17.73 ± 9.18% in DC + PBMC, DC + LNCaP, DC/IL-12 + LNCaP, and DC/IL-15 + LNCaP cultures, respectively. Cytokine-mediated protection of DC from prostate cancer-induced apoptotic death was statistically significant ($P < 0.001$ for both IL-12 and IL-15). Thus, altogether these results suggested that IL-12 and IL-15 protect DC from prostate cancer-induced cell death in vitro.

### CD154 induced increased expression of IL-12 and IL-15 by DC

Since it has been reported that DC produce IL-12 and IL-15 upon stimulation, we have evaluated the possible mechanism of the protective effect of CD154 on DC survival in the prostate cancer microenvironment and examined whether CD154 might increase the expression of IL-12 and IL-15 in cultured DC. CD154 is a known promoter of IL-12 expression on various cells, including DC (Cella et al, 1996; Kelsall et al, 1996). However, its effect on IL-15 expression was not studied yet. Human DC were incubated with human CD154-transfected L-cells. DC were harvested after 24 hours and RT–PCR was performed to evaluate IL-12 and IL-15 mRNA expression. DC incubated with non-transfected L-cells for 24 hours served as controls. We found that CD154-transfected L-cells induced a marked increase in expression of both IL-12 and IL-15 mRNA as compared to controls (Figure 5). For instance, expression of IL-12 p40 in DC co-incubated with non-transfected L-cells (control) was 0.59 (ratio between the densitometry units of the sample band and the corresponded β-actin band), whereas the p40 expression in DC co-incubated with CD154-transfected L-cells was 1.58 (Figure 5A). These results demonstrated that CD40 ligation on cultured human DC increased IL-12 expression up to 270%. Next, IL-15 mRNA expression increased from 0.29 in control DC to 1.02 in DC activated by CD154 (Figure 5B), suggesting that CD40 ligation resulted in stimulation of IL-15 expression up to 350%. Taken together, these data showed that CD40L-mediated activation of DC caused a marked stimulation of IL-12 and IL-15 mRNA expression, which may lead to the increased survival of DC.

### CD154 induced increased expression of the anti-apoptotic protein Bcl-$x_L$

Since it has been shown that CD154-mediated stimulation of B cell was accompanied by an increased expression of anti-apoptotic protein Bcl-$x_L$, we hypothesized that similar mechanisms might be involved in CD154-induced protection of human monocyte-derived DC survival in the prostate cancer microenvironment. To test this hypothesis, we examined Bcl-$x_L$ expression in DC following the stimulation with CD154. Cultured DC were co-incubated with CD154-transfected and control L-cells for 24 hours, harvested and Western blot analysis was performed to detect Bcl-$x_L$ protein expression. We found a marked increase in the expression of Bcl-$x_L$ by DC after the co-incubation with CD154-transfected L-cells, as compared to controls (Figure 6A). For instance, expression of Bcl-$x_L$ in CD154-activated DC reached 1182.44 units compared to 506.89 units in DC co-incubated with non-transfected control L-cells. However, co-incubation of DC with control L-cells caused activation of Bcl-$x_L$ expression by itself in comparison to intact DC (up to 3-fold). This effect is due

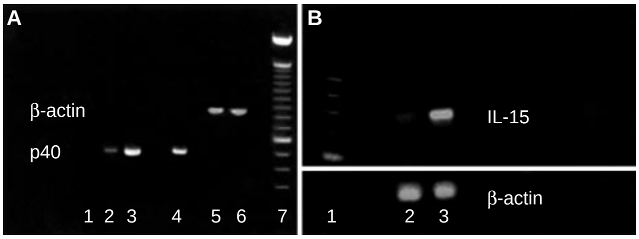

**Figure 5** CD40 ligation on human dendritic cells results in increased expression of IL-12 and IL-15 mRNA. Cultured human PBMC-derived DC were stimulated with CD40L (CD154)-transfected or control (non-transfected) L cells for 24 hours, harvested and washed. The expression of IL-12 mRNA (**A**) and IL-15 mRNA (**B**) was determined as described in Materials and Methods. Analysis of RT–PCR products suggested that CD40 ligation on human DC markedly increased the expression of IL-12 and IL-15 mRNA. Lanes for **A**: 1, DC + L-cells (control); 2, DC + CD154/L-cells; 3, negative control; 4, positive control; 5 and 6, β-actin for 1 and 2, respectively; 7, molecular weight markers. Lanes for **B**: 1, molecular weight markers; 2, DC + L-cells (control); 3, DC + CD154/L-cells

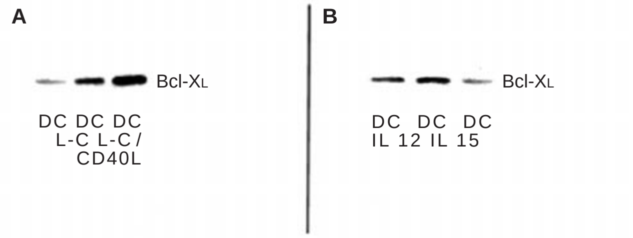

**Figure 6** Activation of human DC with IL-12, IL-15 or CD40L results in increased expression of anti-apoptotic protein Bcl-$x_L$. Cultured human PBMC-derived DC were stimulated with CD40L (CD154)-transfected or control (non-transfected) L-cells (**A**), IL-12 (100 ng/ml) or IL-15 (300 ng/ml) (**B**) for 24 hours, harvested and washed. The expression of Bcl-$x_L$ by DC was determined by Western blot as described in Materials and Methods to a non-specific activation of human DC by murine L-cells. Thus, stimulation of Bcl-$x_L$ expression in DC may be one of the mechanisms responsible for increased survival of CD40L-activated DC in the prostate cancer microenvironment.

### IL-12 and IL-15 increased expression of the anti-apoptotic protein Bcl-$x_L$ by DC

Next, we examined whether cytokine-induced protection of DC from prostate cancer-induced apoptosis might be mediated by an increased expression of anti-apoptotic proteins. Cultured DC were incubated with either *hr*IL-12 (100 ng/ml) or *hr*IL-15 (300 ng/ml) for 24 hours and the expression of Bcl-$x_L$ was determined by a Western blot. The result demonstrated that stimulation of DC with IL-12 or IL-15 in vitro resulted in increased expression of anti-apoptotic protein Bcl-$x_L$ (Figure 6B). For example, expression of Bcl-$x_L$ in control DC corresponded to 163.41 units and was increased to 344.51 units by IL-12 and to 537.17 units by IL-15. Thus, these data suggest that cytokine-induced expression of Bcl-$x_L$ in DC might explain the elevated resistance of DC to prostate cancer-induced apoptosis after stimulation with IL-12, IL-15 or CD40L.

### DISCUSSION

Initiation of a specific antitumour immune response requires presentation of tumour-associated antigen(s) (TAA) to T cells by APC. In fact, it has been well documented that DC, the most potent APC, are capable of recognising, processing and presenting TAA, in turn initiating a specific antitumour immune response (Shurin, 1996). It is also known that infiltration of various tumours with DC confers them favourable prognosis (Becker, 1992; Lotze, 1997). Therefore, it is possible to hypothesize that dysfunction of the DC system may lead to a failure of antitumour immune responses. Prostate cancer was found to be sparse in tumour-infiltrating DC in comparison with benign prostate hyperplasia and normal prostate tissues. The number of DC further decreased in the high-grade prostate carcinomas (Bigotti et al, 1991; Davidson et al, 1997; Troy et al, 1998), which carry the worst prognosis (Eastham and Scardino, 1998). It is possible that active suppression of the host immune system, together with loss or attenuation of class I expression by prostate cancer (Bander et al, 1997) may be responsible for the depressed immunity in prostate cancer patients (Ivshina et al, 1995; Healy et al, 1998; Salgaller et al, 1998). Understanding of prostate cancer immunobiology and, in particular, the immunobiology of DC, will help in the development of effective immunotherapy for this disease.

Decreased number of infiltrating DC in prostate cancer tissue allowed us to speculate on the possibility of active elimination of DC by prostate cancer-derived factors within the tumour microenvironment. In fact, all three prostate cancer cell lines tested in this study, LNCaP, DU-145, and PC-3, demonstrated a significant effect on DC survival, as was assessed by the morphological features, TUNEL staining and Annexin V binding assay. This suggests that prostate cancer might actively eliminate DC from the tumour tissue. Although the observed apoptotic rate of DC seems to be not very high, in the real tumour environment tumour-to-DC ratio is probably several times higher than the one used in our experiments, and apoptotic signals may act for a longer period of time, resulting in complete elimination of DC from prostate cancer tissues. The absence of DC in human prostate cancer tissues, described by Troy et al (1998), may be explained by a high level of prostate cancer-induced DC apoptosis in vivo. However, it is unknown which prostate cancer-derived factors may be involved in this phenomenon. A variety of tumours, including prostate cancer (Stearns and Wang, 1998; Sharma et al, 1999; Stearns et al, 1999; Wang et al, 1999), have been shown to release IL-10, a cytokine that could enhance the spontaneous apoptosis in DC (Ludewig et al, 1995). TGF-β is another candidate cytokine, which has been shown to be released by prostate cancer cells (Burchardt et al, 1999; Wikstrom et al, 1999; Cardillo et al, 2000) and might affect DC as well (Caux et al, 1999; Strobl and Knapp, 1999). It is also possible that prostate cancer cells produce NO (Klotz et al, 1998), which in turn could cause apoptosis in DC. Taken together, these data suggest that prostate cancer-derived factors might cause apoptotic death of DC in the tumour microenvironment. Together with our data, these results provide a new mechanism demonstrating how prostate cancer escapes from immune recognition and raise the next question about the possible mechanisms of DC protection from prostate cancer-induced cell death.

It has been recently reported that CD154 binds to CD40 on DC, leading to their increased survival in cultures (Bjorck et al, 1997; Mackey et al, 1998). This fact allowed us to hypothesize that CD40 ligation on DC may also protect them from prostate cancer-induced apoptosis. To test this hypothesis, we used two methods to stimulate CD40 on DC: (i) adherent L-cells transfected with human CD154 and (ii) specific anti-CD40 antibodies. In both cases, increased resistance of CD40L-activated DC to prostate cancer-induced death was demonstrated. Since we have shown early that LNCaP cells express Fas ligand (CD95L) (Salup et al, 1998) and DC express Fas (Shurin et al, 1999), it is possible that induction of apoptosis in DC by prostate cancer is due to CD95/CD95L interaction. This mechanism remains to be evaluated. Furthermore, CD40 stimulation of DC was shown to counteract CD95/CD95L mediated DC apoptosis in cultures (Bjorck et al, 1997). It is likely that increased expression of anti-apoptotic proteins Bcl-2 (Bjorck et al, 1997) and Bcl-$x_L$ (Figure 6) is involved in DC protection from tumour-induced cell death. Bcl-$x_L$ was effective at rendering B cells resistant to CD95/CD95L-mediated killing (Schneider et al, 1997), and it may have played the same role in our experiments. Further studies are in progress to verify this hypothesis. Interestingly, CD40 stimulation does not lead to increased expression of Bcl-$x_L$ in other types of cells (Sbih-Lammali et al, 1999) suggesting a cell-specific mechanism.

CD154 is a known inducer of IL-12 production (Cella et al, 1996; Kelsall et al, 1996; Nakajima et al, 1998). CD154 is being expressed on T lymphocytes, and CD154/CD40 interaction is extremely important for DC and T cell communication and mutual support. The stimulation of CD40 receptor on DC leads to the increased production of IL-12, which in turn stimulates the expression of CD154 on T cells (Peng et al, 1998), reverse anergy of CD+ 4T cells (Grohmann et al, 1998), and stimulates interferon-γ production by T and NK cells (Kobayashi et al, 1989; Chan et al, 1991; Kubin et al, 1994). As shown in this study, CD40 stimulation also leads to the increased expression of IL-15. IL-15 may serve as an attractant for the T cells during the DC/T cell interaction (Jonuleit et al, 1997). IL-15 also induces T cell proliferation and promotes IL-12 production (Avice et al, 1998). Together with IL-12, IL-15 has been shown to stimulate NK cells to produce interferon-γ, tumour necrosis factor-α and macrophage inhibitory factor-1α (Carson et al, 1995; Bluman et al, 1996). Since both IL-12 and IL-15 are involved in the DC/T cells interaction, promote T cell activity (Heufler et al, 1996; Jonuleit et al, 1997), and increase survival of DC, CD154 stimulation of DC should lead to an increased immunologic response. In fact, using a murine tumour model, we have recently demonstrated that the stable transfection of colon adenocarcinoma cells with CD154 gene resulted in a significant inhibition of tumour growth in vivo, which was accompanied by an increased infiltration of tumour tissues with DC and T cells (Esche et al, 1999).

Thus, IL-12 and IL-15 protected DC from prostate cancer-induced apoptosis. This effect, at least in part, was mediated by the increased expression of the anti-apoptotic protein Bcl-$x_L$ in DC. We believe that CD40 stimulation of DC induces increased expression of Bcl-$x_L$ both directly and indirectly via IL-12 and IL-15 release. Thus, it is likely that IL-12 and IL-15 might regulate DC survival by an autocrine mechanism. These DC regulatory properties of CD154, IL-12 and IL-15 may play an important role during the induction and development of the immune responses to prostate cancer by increasing resistance of DC to prostate cancer-induced cell death. Further work is needed to reveal the mechanism of prostate cancer-induced apoptosis of DC and to develop the most effective methods of DC protection.

In summary, our data demonstrated for the first time that human prostate cancer cells LNCaP, DU-145, and PC-3 cause apoptosis of human DC in vitro. This apoptosis-inducing effect could be partly blocked by the stimulation of DC by CD154, IL-12 or IL-15. Anti-apoptotic protein Bcl-$x_L$ is involved in the protection of DC from prostate cancer-induced apoptosis. Taken together, these findings might serve as a basis for the development of new experimental approaches designed to increase efficacy of DC-based immunotherapies of cancer.

### ACKNOWLEDGEMENTS

This study was supported in part by RO1 CA80126 (MRS), RO1 CA84270 (MRS), Pilot Project Program of the Prostate and Urological Cancer Center of the University of Pittsburgh Cancer Institute (MRS and GP), and The Pittsburgh Foundation for Medical Research (MRS and GP).

### REFERENCES

Akifusa S, Ohguchi M, Koseki T, Nara K, Semba I, Yamato K, Okahashi N, Merino R, Nunez G, Hanada N, Takehara T and Nishihara T (1998) Increase in Bcl-2 level promoted by CD40 ligation correlates with inhibition of B cell apoptosis induced by vacuolar type H(+)-ATPase inhibitor. *Experimental Cell Research* **238:** 82–89

Alam MK, Davison S, Siddiqui N, Norton JD and Murphy JJ (1997) Ectopic expression of Bcl-2, but not Bcl-xL rescues Ramos B cells from Fas-mediated apoptosis. *European Journal of Immunology* **27:** 3485–3491

Arpin C, Dechanet J, Van Kooten C, Merville P, Grouard G, Briere F, Banchereau J and Liu YJ (1995) Generation of memory B cells and plasma cells in vitro. *Science* **268:** 720–722

Avice MN, Demeure CE, Delespesse G, Rubio M, Armant M and Sarfati M (1998) IL-15 promotes IL-12 production by human monocytes via T cell-dependent contact and may contribute to IL-12-mediated IFN-gamma secretion by CD4+ T cells in the absence of TCR ligation. *Journal of Immunology* **161:** 3408–3415

Bander NH, Yao D, Liu H, Chen YT, Steiner M, Zuccaro W and Moy P (1997) MHC class I and II expression in prostate carcinoma and modulation by interferon-alpha and -gamma. *Prostate* **33:** 233–239

Becker Y (1992) Anticancer role of dendritic cells (DC) in human and experimental cancers – a review. *Anticancer Research* **12:** 511–520

Bellone G and Trinchieri G (1994) Dual stimulatory and inhibitory effect of NK cell stimulatory factor/IL-12 on human hematopoiesis. *Journal of Immunology* **153:** 930–937

Bigotti G, Coli A and Castagnola D (1991) Distribution of Langerhans cells and HLA class II molecules in prostatic carcinomas of different histopathological grade. *Prostate* **19:** 73–87

Bjorck P, Banchereau J and Flores-Romo L (1997) CD40 ligation counteracts Fas-induced apoptosis of human dendritic cells. *International Immunology* **9:** 365–372

Bluman EM, Bartynski KJ, Avalos BR and Caligiuri MA (1996) Human natural killer cells produce abundant macrophage inflammatory protein-1 alpha in response to monocyte-derived cytokines. *Journal of Clinical Investigation* **97:** 2722–2727

Bulfone-Paus S, Ungureanu D, Pohl T, Lindner G, Paus R, Ruckert R, Krause H, and Kunzendorf U (1997) Interleukin-15 protects from lethal apoptosis in vivo. *Nature Medicine* **3:** 1124–1128

Burchardt T, Burchardt M, Chen MW, Cao Y, de la Taille A, Shabsigh A, Hayek O, Dorai T and Buttyan R (1999) Transdifferentiation of prostate cancer cells to a neuroendocrine cell phenotype in vitro and in vivo. *J Urol* **162:** 1800–1805

Cardillo MR, Petrangeli E, Perracchio L, Salvatori L, Ravenna L and Di Silverio F. (2000). Transforming growth factor-beta expression in prostate neoplasia [In Process Citation]. *Anal Quant Cytol Histol* **22:** 1–10

Carson WE, Ross ME, Baiocchi RA, Marien MJ, Boiani N, Grabstein K and Caligiuri MA (1995) Endogenous production of interleukin 15 by activated human monocytes is critical for optimal production of interferon-gamma by natural killer cells in vitro. *Journal of Clinical Investigation* **96:** 2578–2582

Carson WE, Fehniger TA, Haldar S, Eckhert K, Lindemann MJ, Lai CF, Croce CM, Baumann H and Caligiuri MA (1997) A potential role for interleukin-15 in the regulation of human natural killer cell survival. *Journal of Clinical Investigation* **99:** 937–943

Caux C, Massacrier C, Dubois B, Valladeau J, Dezutter-Dambuyant C, Durand I, Schmitt D and Saeland S (1999) Respective involvement of TGF-beta and IL-4 in the development of Langerhans cells and non-Langerhans dendritic cells from CD34+ progenitors. *J Leukoc Biol* **66:** 781–791

© 2000 Cancer Research Campaign

Cella M, Scheidegger D, Palmer-Lehmann K, Lane P, Lanzavecchia A and Alber G (1996) Ligation of CD40 on dendritic cells triggers production of high levels of interleukin-12 and enhances T cell stimulatory capacity: T-T help via APC activation. *Journal of Experimental Medicine* **184:** 747–752

Chan SH, Perussia B, Gupta JW, Kobayashi M, Pospisil M, Young HA, Wolf SF, Young D, Clark SC and Trinchieri G (1991) Induction of interferon gamma production by natural killer cell stimulatory factor: characterization of the responder cells and synergy with other inducers. *Journal of Experimental Medicine* **173:** 869–879

Davidson PJT, Troy AJ, Atkinson CH and DNJ H 1997 Characterization of the dendritic cell response to the carcinoma of the prostate. In: *AUA 92nd Annual Meeting*, New Orleans, p. 370

Eastham JA and Scardino PT 1998 Radical prostatectomy. In: *Campbell's Urology*. PC Walsh, AB Retik, ED Vaughan and AJ Wein eds. WB Saunders Company, pp 2547–2564

Esche C, Gambotto A, Satoh Y, Gerein V, Robbins PD, Watkins SC, Lotze MT and Shurin MR (1999a) CD154 inhibits tumor-induced apoptosis in dendritic cells and tumor growth. *Eur J Immunol* **29:** 2148–2155

Esche C, Lokshin A, Shurin GV, Gastman BR, Rabinowich H, Watkins SC, Lotze MT and Shurin MR (1999b) Tumor's other immune targets: dendritic cells [In Process Citation]. *J Leukoc Biol* **66:** 336–344.

Grohmann U, Fioretti MC, Bianchi R, Belladonna ML, Ayroldi E, Surace D, Silla S and Puccetti P (1998) Dendritic cells, interleukin 12, and CD4+ lymphocytes in the initiation of class I-restricted reactivity to a tumor/self peptide. *Critical Reviews in Immunology* **18:** 87–98

Healy CG, Simons JW, Carducci MA, DeWeese TL, Bartkowski M, Tong KP and Bolton WE (1998) Impaired expression and function of signal-transducing zeta chains in peripheral T cells and natural killer cells in patients with prostate cancer. *Cytometry* **32:** 109–119

Herr HW (1980) Suppressor cells in immunodepressed bladder and prostate cancer patients. *Journal of Urology* **123:** 635–639

Heufler C, Koch F, Stanzl U, Topar G, Wysocka M, Trinchieri G, Enk A, Steinman RM, Romani N and Schuler G (1996) Interleukin-12 is produced by dendritic cells and mediates T helper 1 development as well as interferon-gamma production by T helper 1 cells. *European Journal of Immunology* **26:** 659–668

Ivshina AV, Zhumagazin Zh D, Zabotina TN, Matveev BP and Kadagidze ZG (1995) [Effect of the spread of the process and treatment on the phenotype of peripheral blood lymphocytes in patients with prostatic cancer]. *Urologiia i Nefrologiia*: 36–38.

Jonuleit H, Wiedemann K, Muller G, Degwert J, Hoppe U, Knop J and Enk AH (1997) Induction of IL-15 messenger RNA and protein in human blood-derived dendritic cells: a role for IL-15 in attraction of T cells. *Journal of Immunology* **158:** 2610–2615

Kelsall BL, Stuber E, Neurath M and Strober W (1996) Interleukin-12 production by dendritic cells. The role of CD40-CD40L interactions in Th1 T-cell responses. *Annals of the New York Academy of Sciences* **795:** 116–126

Kerr JF, Wyllie AH and Currie AR (1972) Apoptosis: a basic biological phenomenon with wide-ranging implications in tissue kinetics. *British Journal of Cancer* **26:** 239–257

Klotz T, Bloch W, Volberg C, Engelmann U and Addicks K (1998) Selective expression of inducible nitric oxide synthase in human prostate carcinoma. *Cancer* **82:** 1897–1903

Kobayashi M, Fitz L, Ryan M, Hewick RM, Clark SC, Chan S, Loudon R, Sherman F, Perussia B and Trinchieri G (1989) Identification and purification of natural killer cell stimulatory factor (NKSF), a cytokine with multiple biologic effects on human lymphocytes. *Journal of Experimental Medicine* **170:** 827–845

Kubin M, Kamoun M and Trinchieri G (1994) Interleukin 12 synergizes with B7/CD28 interaction in inducing efficient proliferation and cytokine production of human T cells. *Journal of Experimental Medicine* **180:** 211–222

Landis SH, Murray T, Bolden S and Wingo PA (1999) Cancer statistics, 1999. *Ca: a Cancer Journal for Clinicians* **49:** 8–31

Lorenz HM, Hieronymus T, Grunke M, Manger B and Kalden JR (1997) Differential role for IL-2 and IL-15 in the inhibition of apoptosis in short-term activated human lymphocytes. *Scandinavian Journal of Immunology* **45:** 660–669

Lotze MT (1997) Getting to the source: dendritic cells as therapeutic reagents for the treatment of patients with cancer. *Annals of Surgery* **226:** 1–5

Ludewig B, Graf D, Gelderblom HR, Becker Y, Kroczek RA and Pauli G (1995) Spontaneous apoptosis of dendritic cells is efficiently inhibited by TRAP (CD40-ligand) and TNF-alpha, but strongly enhanced by interleukin-10. *Eur J Immunol* **25:** 1943–1950

Mackey MF, Gunn JR, Maliszewsky C, Kikutani H, Noelle RJ and Barth RJ Jr (1998) Dendritic cells require maturation via CD40 to generate protective antitumor immunity. *Journal of Immunology* **161:** 2094–2098

Nakajima A, Kodama T, Morimoto S, Azuma M, Takeda K, Oshima H, Yoshino S, Yagita H and Okumura K (1998) Antitumor effect of CD40 ligand: elicitation of local and systemic antitumor responses by IL-12 and B7. *Journal of Immunology* **161:** 1901–1907

Ning ZQ, Norton JD, Li J and Murphy JJ (1996) Distinct mechanisms for rescue from apoptosis in Ramos human B cells by signaling through CD40 and interleukin-4 receptor: role for inhibition of an early response gene, Berg36. *European Journal of Immunology* **26:** 2356–2363

Peng X, Remacle JE, Kasran A, Huylebroeck D and Ceuppens JL (1998) IL-12 up-regulates CD40 ligand (CD154) expression on human T cells. *Journal of Immunology* **160:** 1166–1172

Ploemacher RE, van Soest PL, Voorwinden H and Boudewijn A (1993) Interleukin-12 synergizes with interleukin-3 and steel factor to enhance recovery of murine hemopoietic stem cells in liquid culture. *Leukemia* **7:** 1381–1388

Salgaller ML, Lodge PA, McLean JG, Tjoa BA, Loftus DJ, Ragde H, Kenny GM, Rogers M, Boynton AL and Murphy GP (1998a) Report of immune monitoring of prostate cancer patients undergoing T-cell therapy using dendritic cells pulsed with HLA-A2-specific peptides from prostate-specific membrane antigen (PSMA). *Prostate* **35:** 144–151

Salgaller ML, Tjoa BA, Lodge PA, Ragde H, Kenny G, Boynton A and Murphy GP (1998b) Dendritic cell-based immunotherapy of prostate cancer. *Critical Reviews in Immunology* **18:** 109–119

Salup RR, Deng DH and Baar J (1998) Analysis of Fas expression and growth of prostate cancer cells. *Proceed. AACR* **39:** 580–581

Sbih-Lammali F, Clausse B, Ardila-Osorio H, Guerry R, Talbot M, Havouis S, Ferradini L, Bosq J, Tursz T and Busson P (1999) Control of apoptosis in Epstein Barr virus-positive nasopharyngeal carcinoma cells: opposite effects of CD95 and CD40 stimulation. *Cancer Research* **59:** 924–930

Schneider TJ, Grillot D, Foote LC, Nunez GE and Rothstein TL (1997) Bcl-x protects primary B cells against Fas-mediated apoptosis. *Journal of Immunology* **159:** 4834–4839

Sharma N, Luo J, Kirschmann DA, O'Malley Y, Robbins ME, Akporiaye ET, Lubaroff DM, Heidger PM and Hendrix MJ (1999) A novel immunological model for the study of prostate cancer. *Cancer Res* **59:** 2271–2276

Shurin MR (1996) Dendritic cells presenting tumor antigen. *Cancer Immunology, Immunotherapy* **43:** 158–164

Shurin MR (1999) Regulation of dendropoiesis in cancer. *Clin Immunol Newsletter* **19:** 135–139

Shurin MR, Esche C, Lokshin A and Lotze MT (1999) Apoptosis in Dendritic Cells. In: *Dendritic Cells: Biology and Clinical Applications*. MT Lotze and AW Thomson eds. Academic Press, San Diego, pp 673–692

Stearns ME and Wang M (1998) Antimetastatic and antitumor activities of interleukin 10 in transfected human prostate PC-3 ML clones: Orthotopic growth in severe combined immunodeficient mice. *Clin Cancer Res* **4:** 2257–2263

Stearns ME, Rhim J and Wang M (1999) Interleukin 10 (IL-10) inhibition of primary human prostate cell-induced angiogenesis: IL-10 stimulation of tissue inhibitor of metalloproteinase-1 and inhibition of matrix metalloproteinase (MMP)- 2/MMP-9 secretion. *Clin Cancer Res* **5:** 189–196

Steinman RM and Cohn ZA (1973) Identification of a novel cell type in peripheral lymphoid organs of mice. I. Morphology, quantitation, tissue distribution. *Journal of Experimental Medicine* **137:** 1142–1162

Stene MA, Babajanians M, Bhuta S and Cochran AJ (1988) Quantitative alterations in cutaneous Langerhans cells during the evolution of malignant melanoma of the skin. *Journal of Investigative Dermatology* **91:** 125–128

Strobl H and Knapp W (1999) TGF-betal regulation of dendritic cells. *Microbes Infect* **1:** 1283–1290

Toriyama K, Wen DR, Paul E and Cochran AJ (1993) Variations in the distribution, frequency, and phenotype of Langerhans cells during the evolution of malignant melanoma of the skin. *Journal of Investigative Dermatology* **100:** 269S–273S

Troy A, Davidson P, Atkinson C and Hart D (1998) Phenotypic characterisation of the dendritic cell infiltrate in prostate cancer. *Journal of Urology* **160:** 214–219

Wang M, Liu A, Garcia FU, Rhim JS and Stearns ME (1999) Growth of HPV-18 immortalized human prostatic intraepithelial neoplasia cell lines. Influence of IL-10, follistatin, activin-A, and DHT. *Int J Oncol* **14:** 1185–1195

Wikstrom P, Lindh G, Bergh A and Damber JE (1999) Alterations of transforming growth factor beta1 (TGF-beta1) and TGFbeta receptor expressions with progression in Dunning rat prostatic adenocarcinoma sublines. *Urol Res* **27:** 185–193

© 2000 Cancer Research Campaign 
